# Tetra­kis(3-cyano­pyridine-κ*N* ^1^)bis­(thio­cyanato-κ*N*)cobalt(II) 1,4-dioxane disolvate

**DOI:** 10.1107/S1600536811051087

**Published:** 2011-11-30

**Authors:** Stephan Diehr, Susanne Wöhlert, Jan Boeckmann, Christian Näther

**Affiliations:** a Institut für Anorganische Chemie, Christian-Albrechts-Universität Kiel, Max-Eyth-Strasse 2, 24098 Kiel, Germany

## Abstract

In the crystal structure of the title compound, {[Co(NCS)_2_(C_6_H_4_N_2_)_4_]·2C_4_H_8_O_2_}, the Co^II^ cations are octa­hedrally coordinated by two terminal *N*-bonded thio­cyanate anions and four *N*-bonded 3-cyano­pyridine ligands. The asymmetric unit consists of one Co^II^ cation, which is located on a special position with site symmetry 2/*m*, one thio­cyanate anion and one dioxane mol­ecule, located on a crystallographic mirror plane, as well as one 3-cyano­pyridine ligand in a general position. The crystal structure consists of discrete complexes of [Co(NCS)_2_(3-cyano­pyridine)_4_], as well as two non-coordinating 1,4-dioxane solvent mol­ecules which are disordered due to symmetry.

## Related literature

For related structures, see: Kilkenny & Nassimbeni (2001 ▶). For background to this work, see: Boeckmann & Näther (2010 ▶, 2011 ▶); Wöhlert *et al.* (2011 ▶). For a description of the Cambridge Structural Database, see: Allen (2002 ▶).
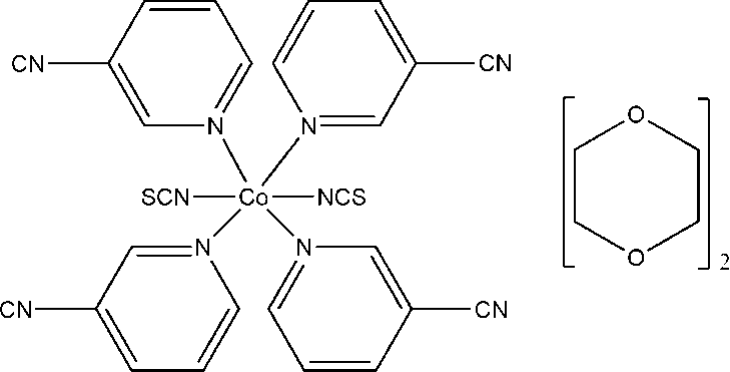


## Experimental

### 

#### Crystal data

[Co(NCS)_2_(C_6_H_4_N_2_)_4_]·2C_4_H_8_O_2_
*M* *_r_* = 767.75
Monoclinic, 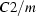

*a* = 15.5222 (6) Å
*b* = 14.1865 (7) Å
*c* = 10.0762 (4) Å
β = 124.454 (3)°
*V* = 1829.61 (14) Å^3^
*Z* = 2
Mo *K*α radiation
μ = 0.64 mm^−1^
*T* = 293 K
0.12 × 0.10 × 0.08 mm

#### Data collection

Stoe IPDS-2 diffractometer
Absorption correction: numerical (*X-RED32* and *X-SHAPE*; Stoe & Cie, 2008) ▶ *T*_min_ = 0.911, *T*_max_ = 0.941
14354 measured reflections
2271 independent reflections
1997 reflections with *I* > 2σ(*I*)
*R*_int_ = 0.061

#### Refinement

*R*[*F* ^2^ > 2σ(*F* ^2^)] = 0.044
*wR*(*F* ^2^) = 0.098
*S* = 1.17
2271 reflections
143 parameters
H-atom parameters constrained
Δρ_max_ = 0.26 e Å^−3^
Δρ_min_ = −0.27 e Å^−3^

### 

Data collection: *X-AREA* (Stoe & Cie, 2008) ▶; cell refinement: *X-AREA*; data reduction: *X-AREA*; program(s) used to solve structure: *SHELXS97* (Sheldrick, 2008 ▶); program(s) used to refine structure: *SHELXL97* (Sheldrick, 2008 ▶); molecular graphics: *XP* in *SHELXTL* (Sheldrick, 2008 ▶) and *DIAMOND* (Brandenburg, 2010 ▶); software used to prepare material for publication: *XCIF* in *SHELXTL*.

## Supplementary Material

Crystal structure: contains datablock(s) I, global. DOI: 10.1107/S1600536811051087/wm2564sup1.cif

Structure factors: contains datablock(s) I. DOI: 10.1107/S1600536811051087/wm2564Isup2.hkl

Additional supplementary materials:  crystallographic information; 3D view; checkCIF report

## Figures and Tables

**Fig. 1.**
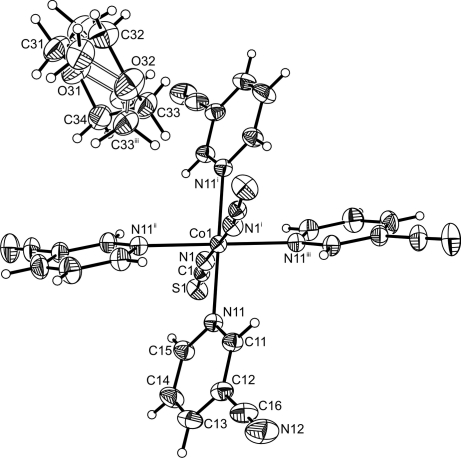
: The molecular moieties of the crystal structure of the title compound with atom labelling and displacement ellipsoids drawn at the 30% probability level. The disorder of the dioxane ligand is shown by full and open bonds. [Symmetry codes: i: -x + 1, -y + 1, -z + 1; ii: -x + 1, y, -z + 1; iii: x, -y + 1, z.]

**Fig. 2.**
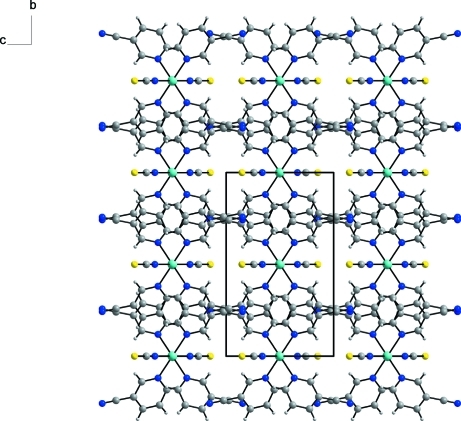
: Crystal structure of the title compound with view along the crystallographic a-axis. The non-coordinated 1,4-dioxane were omitted for clarity.

## supplementary crystallographic information

### Comment

Recently, we became interested in new transition metal thiocyanato coordination polymers with terminal thiocyanate anions that can be used as precursors in thermal decomposition reactions in order to prepare new coordination compounds in which the metal cations are linked by the anionic ligands (Boeckmann & Näther, 2010, 2011; Wöhlert *et al.*, 2011). In our ongoing investigation in this field we have reacted cobalt(II) thiocyanate and 3-cyanopyridine in dioxane. In this reaction light-red single crystals of the title compound were obtained, which were characterized by single-crystal X-ray diffraction.

In the crystal structure of the title compound, [Co(NCS)_2_(C_6_H_4_N_2_)_4_]^.^2(C_4_H_8_O_2_), the cobalt(II) cations are coordinated by two terminal *N*-bonded thiocyanate anions and by four 3-cyanopyridine ligands into discrete complexes which are located on special positions with site symmetry 2/*m* (Fig. 1). The octahedral coordination sphere of the cobalt(II) cations is slightly distorted with distances in the range of 2.036 (2) Å to 2.2451 (16) Å. The angles around the cobalt(II) cations range from 89.67 (6)° to 180 °. The discrete complexes are stacked into columns that elongate in the direction of the *c*-axis (Fig. 2). From this arrangement channels are formed in which the disordered dioxane molecules are located. It should be noted that according to a search in the CCDC database (CONQUEST Ver. 1.13.2011; Allen, 2002) discrete complexes based on cobalt(II) thiocyanate and 3-cyanopyridine with solvate molecules (i.e. ethanol and dichloromethane) have already been reported (Kilkenny & Nassimbeni, 2001).

### Experimental

Cobalt(II) thiocyanate and 3-cyanopyridine were obtained from Alfa Aesar, and 1,4-dioxane from Sigma Aldrich. 0.25 mmol (44.0 mg) Co(NCS)_2_^.^*x*H_2_O, 0.50 mmol (52.1 mg) 3-cyanopyridine and 1.5 ml 1,4-dioxane were reacted in a closed snap-vial without stirring. After the mixture has been standing for several days at room temperature light-red single crystals of the title compound were obtained on slow evaporation of the solvent.

### Refinement

All non-hydrogen atoms were refined anisotropically. All H atoms were positioned with idealized geometry and were refined using a riding model with *U*_eq_(H) = 1.2 *U*_eq_(C). The dioxane molecules are disordered around crystallographic mirror planes. On refinement of this structure in space group *C*2 or *Cm* the disorder remains constant and therefore space group *C*2/*m* was selected. Moreover, analysis of the reciprocal space gave no hints for super structure reflections.

### Crystal data

**Table:**

[Co(NCS)_2_(C_6_H_4_N_2_)_4_]·2C_4_H_8_O_2_	*F*(000) = 794
*M**_r_* = 767.75	*D*_x_ = 1.394 Mg m^−^^3^
Monoclinic, *C*2/*m*	Mo *K*α radiation, λ = 0.71073 Å
Hall symbol: -C 2y	Cell parameters from 14354 reflections
*a* = 15.5222 (6) Å	θ = 2.1–28.0°
*b* = 14.1865 (7) Å	µ = 0.64 mm^−^^1^
*c* = 10.0762 (4) Å	*T* = 293 K
β = 124.454 (3)°	Block, light-red
*V* = 1829.61 (14) Å^3^	0.12 × 0.10 × 0.08 mm
*Z* = 2	

### Data collection

**Table:**

Stoe IPDS-2 diffractometer	2271 independent reflections
Radiation source: fine-focus sealed tube	1997 reflections with *I* > 2σ(*I*)
graphite	*R*_int_ = 0.061
ω scans	θ_max_ = 28.0°, θ_min_ = 2.1°
Absorption correction: numerical (*X-RED32* and *X-SHAPE*; Stoe & Cie, 2008)	*h* = −20→20
*T*_min_ = 0.911, *T*_max_ = 0.941	*k* = −18→18
14354 measured reflections	*l* = −13→13

### Refinement

**Table:**

Refinement on *F*^2^	Primary atom site location: structure-invariant direct methods
Least-squares matrix: full	Secondary atom site location: difference Fourier map
*R*[*F*^2^ > 2σ(*F*^2^)] = 0.044	Hydrogen site location: inferred from neighbouring sites
*wR*(*F*^2^) = 0.098	H-atom parameters constrained
*S* = 1.17	*w* = 1/[σ^2^(*F*_o_^2^) + (0.0397*P*)^2^ + 0.8865*P*] where *P* = (*F*_o_^2^ + 2*F*_c_^2^)/3
2271 reflections	(Δ/σ)_max_ < 0.001
143 parameters	Δρ_max_ = 0.26 e Å^−^^3^
0 restraints	Δρ_min_ = −0.27 e Å^−^^3^

### Special details

**Table:**

Geometry. All e.s.d.'s (except the e.s.d. in the dihedral angle between two l.s. planes) are estimated using the full covariance matrix. The cell e.s.d.'s are taken into account individually in the estimation of e.s.d.'s in distances, angles and torsion angles; correlations between e.s.d.'s in cell parameters are only used when they are defined by crystal symmetry. An approximate (isotropic) treatment of cell e.s.d.'s is used for estimating e.s.d.'s involving l.s. planes.
Refinement. Refinement of *F*^2^ against ALL reflections. The weighted *R*-factor *wR* and goodness of fit *S* are based on *F*^2^, conventional *R*-factors *R* are based on *F*, with *F* set to zero for negative *F*^2^. The threshold expression of *F*^2^ > σ(*F*^2^) is used only for calculating *R*-factors(gt) *etc*. and is not relevant to the choice of reflections for refinement. *R*-factors based on *F*^2^ are statistically about twice as large as those based on *F*, and *R*- factors based on ALL data will be even larger.

### Fractional atomic coordinates and isotropic or equivalent isotropic displacement parameters (Å^2^)

**Table:**

	*x*	*y*	*z*	*U*_iso_*/*U*_eq_	Occ. (<1)
Co1	0.5000	0.5000	0.5000	0.03493 (15)	
S1	0.12527 (6)	0.5000	0.14723 (11)	0.0599 (2)	
N1	0.34226 (17)	0.5000	0.3366 (3)	0.0451 (5)	
N11	0.48617 (12)	0.61900 (11)	0.63377 (18)	0.0432 (4)	
N12	0.7165 (2)	0.7574 (2)	1.1532 (3)	0.0901 (8)	
C1	0.2515 (2)	0.5000	0.2576 (3)	0.0391 (5)	
C11	0.56168 (16)	0.63928 (14)	0.7862 (2)	0.0470 (4)	
H11	0.6154	0.5960	0.8449	0.056*	
C12	0.56356 (18)	0.72207 (16)	0.8610 (3)	0.0531 (5)	
C13	0.4847 (2)	0.78700 (17)	0.7760 (3)	0.0665 (6)	
H13	0.4844	0.8432	0.8233	0.080*	
C14	0.4063 (2)	0.76611 (18)	0.6192 (3)	0.0696 (7)	
H14	0.3516	0.8082	0.5583	0.084*	
C15	0.40956 (17)	0.68248 (16)	0.5528 (3)	0.0539 (5)	
H15	0.3559	0.6695	0.4465	0.065*	
C16	0.6490 (2)	0.74119 (19)	1.0243 (3)	0.0675 (7)	
O31	0.6959 (2)	0.5000	0.1030 (3)	0.0958 (10)	
C31	0.8027 (5)	0.5305 (4)	0.1665 (7)	0.084 (2)	0.50
H31A	0.8122	0.5958	0.1966	0.101*	0.50
H31B	0.8183	0.5213	0.0878	0.101*	0.50
C32	0.8720 (5)	0.4714 (5)	0.3113 (7)	0.087 (2)	0.50
H32A	0.9443	0.4792	0.3518	0.104*	0.50
H32B	0.8535	0.4063	0.2835	0.104*	0.50
O32	0.8526 (3)	0.5000	0.4305 (3)	0.0994 (11)	
C33	0.7480 (5)	0.4743 (4)	0.3704 (6)	0.083 (2)	0.50
H33A	0.7341	0.4862	0.4504	0.100*	0.50
H33B	0.7372	0.4085	0.3439	0.100*	0.50
C34	0.6764 (5)	0.5311 (4)	0.2226 (7)	0.0811 (18)	0.50
H34A	0.6047	0.5214	0.1842	0.097*	0.50
H34B	0.6927	0.5968	0.2454	0.097*	0.50

### Atomic displacement parameters (Å^2^)

**Table:**

	*U*^11^	*U*^22^	*U*^33^	*U*^12^	*U*^13^	*U*^23^
Co1	0.0262 (2)	0.0399 (3)	0.0286 (2)	0.000	0.00942 (18)	0.000
S1	0.0296 (4)	0.0637 (5)	0.0687 (5)	0.000	0.0173 (3)	0.000
N1	0.0295 (10)	0.0522 (13)	0.0391 (11)	0.000	0.0108 (9)	0.000
N11	0.0391 (8)	0.0451 (8)	0.0415 (8)	0.0013 (7)	0.0205 (7)	−0.0009 (6)
N12	0.0892 (17)	0.0930 (18)	0.0677 (14)	−0.0095 (14)	0.0321 (13)	−0.0358 (13)
C1	0.0380 (13)	0.0406 (13)	0.0349 (12)	0.000	0.0183 (10)	0.000
C11	0.0435 (10)	0.0493 (11)	0.0428 (9)	0.0018 (8)	0.0212 (8)	−0.0045 (8)
C12	0.0555 (12)	0.0534 (12)	0.0550 (11)	−0.0057 (10)	0.0340 (10)	−0.0120 (9)
C13	0.0743 (17)	0.0518 (12)	0.0812 (16)	0.0049 (11)	0.0487 (14)	−0.0127 (11)
C14	0.0649 (15)	0.0585 (14)	0.0782 (16)	0.0219 (12)	0.0362 (13)	0.0052 (12)
C15	0.0458 (11)	0.0549 (12)	0.0533 (11)	0.0096 (9)	0.0233 (9)	0.0027 (9)
C16	0.0716 (16)	0.0665 (15)	0.0647 (14)	−0.0050 (12)	0.0387 (13)	−0.0232 (12)
O31	0.0758 (19)	0.153 (3)	0.0465 (13)	0.000	0.0271 (14)	0.000
C31	0.093 (4)	0.110 (6)	0.067 (3)	0.005 (3)	0.056 (3)	0.003 (3)
C32	0.066 (3)	0.109 (7)	0.074 (3)	0.006 (3)	0.033 (3)	−0.004 (3)
O32	0.087 (2)	0.144 (3)	0.0476 (14)	0.000	0.0270 (14)	0.000
C33	0.109 (4)	0.090 (6)	0.065 (3)	−0.001 (3)	0.058 (3)	0.004 (3)
C34	0.083 (4)	0.089 (5)	0.082 (3)	0.011 (3)	0.053 (3)	0.015 (3)

### Geometric parameters (Å, °)

**Table:**

Co1—N1^i^	2.036 (2)	C15—H15	0.9300
Co1—N1	2.036 (2)	O31—C31^iii^	1.464 (7)
Co1—N11^i^	2.2451 (16)	O31—C31	1.464 (7)
Co1—N11	2.2451 (16)	O31—C34	1.466 (6)
Co1—N11^ii^	2.2451 (16)	O31—C34^iii^	1.466 (6)
Co1—N11^iii^	2.2451 (16)	C31—C32	1.488 (8)
S1—C1	1.615 (3)	C31—H31A	0.9599
N1—C1	1.162 (3)	C31—H31B	0.9599
N11—C11	1.334 (2)	C32—O32	1.451 (6)
N11—C15	1.339 (3)	C32—H32A	0.9600
N12—C16	1.140 (3)	C32—H32B	0.9601
C11—C12	1.387 (3)	O32—C33	1.423 (7)
C11—H11	0.9300	O32—C33^iii^	1.423 (7)
C12—C13	1.376 (3)	O32—C32^iii^	1.451 (6)
C12—C16	1.439 (3)	C33—C34	1.493 (7)
C13—C14	1.375 (4)	C33—H33A	0.9600
C13—H13	0.9300	C33—H33B	0.9600
C14—C15	1.377 (3)	C34—H34A	0.9600
C14—H14	0.9300	C34—H34B	0.9599
			
N1^i^—Co1—N1	180.00 (10)	N11—C15—C14	123.2 (2)
N1^i^—Co1—N11^i^	90.33 (6)	N11—C15—H15	118.4
N1—Co1—N11^i^	89.67 (6)	C14—C15—H15	118.4
N1^i^—Co1—N11	89.67 (6)	N12—C16—C12	179.2 (3)
N1—Co1—N11	90.33 (6)	C31—O31—C34	105.1 (4)
N11^i^—Co1—N11	180.0	C31^iii^—O31—C34^iii^	105.1 (4)
N1^i^—Co1—N11^ii^	90.33 (6)	O31—C31—C32	105.9 (4)
N1—Co1—N11^ii^	89.67 (6)	O31—C31—H31A	110.9
N11^i^—Co1—N11^ii^	97.52 (8)	C32—C31—H31A	109.7
N11—Co1—N11^ii^	82.48 (8)	O31—C31—H31B	110.6
N1^i^—Co1—N11^iii^	89.67 (6)	C32—C31—H31B	110.7
N1—Co1—N11^iii^	90.33 (6)	H31A—C31—H31B	109.0
N11^i^—Co1—N11^iii^	82.48 (8)	O32—C32—C31	106.3 (4)
N11—Co1—N11^iii^	97.52 (8)	O32—C32—H32A	110.8
N11^ii^—Co1—N11^iii^	180.00 (5)	C31—C32—H32A	112.0
C1—N1—Co1	172.6 (2)	O32—C32—H32B	110.1
C11—N11—C15	117.11 (18)	C31—C32—H32B	109.0
C11—N11—Co1	122.24 (13)	H32A—C32—H32B	108.7
C15—N11—Co1	119.56 (13)	C33^iii^—O32—C32^iii^	107.3 (4)
N1—C1—S1	179.8 (2)	C33—O32—C32	107.3 (4)
N11—C11—C12	122.9 (2)	O32—C33—C34	108.2 (4)
N11—C11—H11	118.6	O32—C33—H33A	110.0
C12—C11—H11	118.6	C34—C33—H33A	110.5
C13—C12—C11	119.4 (2)	O32—C33—H33B	110.2
C13—C12—C16	120.3 (2)	C34—C33—H33B	109.4
C11—C12—C16	120.2 (2)	H33A—C33—H33B	108.5
C14—C13—C12	117.9 (2)	O31—C34—C33	106.0 (4)
C14—C13—H13	121.1	O31—C34—H34A	111.0
C12—C13—H13	121.1	C33—C34—H34A	111.3
C13—C14—C15	119.5 (2)	O31—C34—H34B	109.9
C13—C14—H14	120.2	C33—C34—H34B	109.8
C15—C14—H14	120.2	H34A—C34—H34B	108.8

Symmetry codes: (i) −*x*+1, −*y*+1, −*z*+1; (ii) −*x*+1, *y*, −*z*+1; (iii) *x*, −*y*+1, *z*.

## References

[bb1] Allen, F. H. (2002). *Acta Cryst.* B**58**, 380–388. 10.1107/s0108768102003890 12037359

[bb2] Boeckmann, J. & Näther, C. (2010). *Dalton Trans.* **39**, 11019–11026. 10.1039/c0dt00904k 20949144

[bb3] Boeckmann, J. & Näther, C. (2011). *Chem. Commun.* **47**, 7104–7106. 10.1039/c1cc12273h 21617809

[bb4] Brandenburg, K. (2010). *DIAMOND* Crystal Impact GbR, Bonn, Germany.

[bb5] Kilkenny, M. L. & Nassimbeni, L. R. (2001). *J. Chem. Soc. Dalton Trans.* pp. 3065–3068.

[bb6] Sheldrick, G. M. (2008). *Acta Cryst.* A**64**, 112–122. 10.1107/S0108767307043930 18156677

[bb7] Stoe & Cie (2008). *X-AREA*, *X-RED32* and *X-SHAPE* Stoe & Cie, Darmstadt, Germany.

[bb8] Wöhlert, S., Boeckmann, J., Wriedt, M. & Näther, C. (2011). *Angew. Chem. Int. Ed.* **50**, 6920–6923. 10.1002/anie.201007899 21671312

